# Supplementary material for: Single-atom platinum with asymmetric coordination environment on fully conjugated covalent organic framework for efficient electrocatalysis
Source: Nat Commun. 2024 Mar 22;15:2556. doi: 10.1038/s41467-024-46872-x (PMC10960042; doi:10.1038/s41467-024-46872-x)
Supplement: Supplementary file 1 — Supplementary Information [file 41467_2024_46872_MOESM1_ESM.pdf]

Supplementary Information

Single-Atom Platinum with Asymmetric Coordination Environment on Fully Conjugated Covalent Organic Framework for Efficient Electrocatalysis

Zhang et al.

Supplementary Discussion

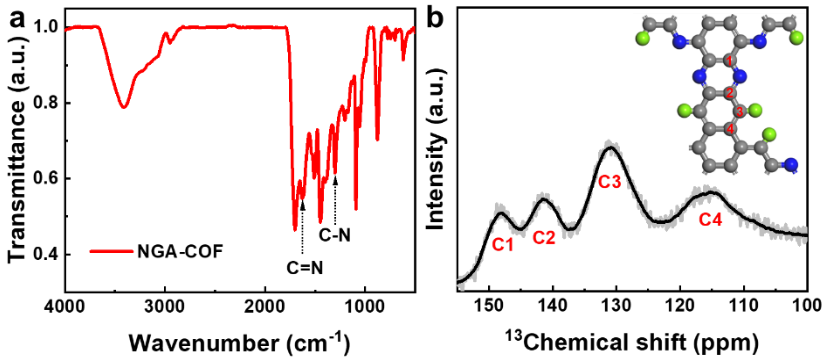

Supplementary Fig. 1. a FTIR and **b** solid-state CP-MAS ^{13}C NMR spectra of NGA-COF.

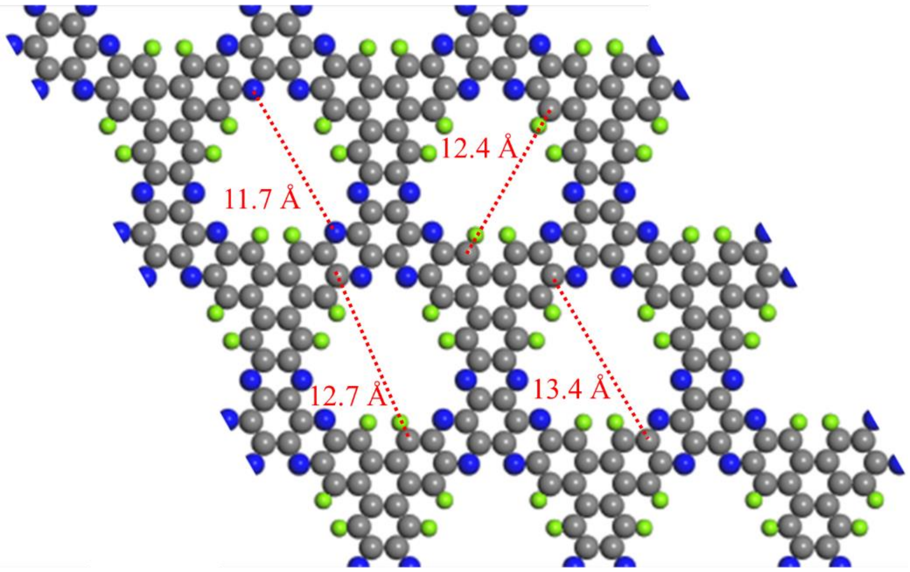

Supplementary Fig. 2. Simulated pore size of NGA-COF.

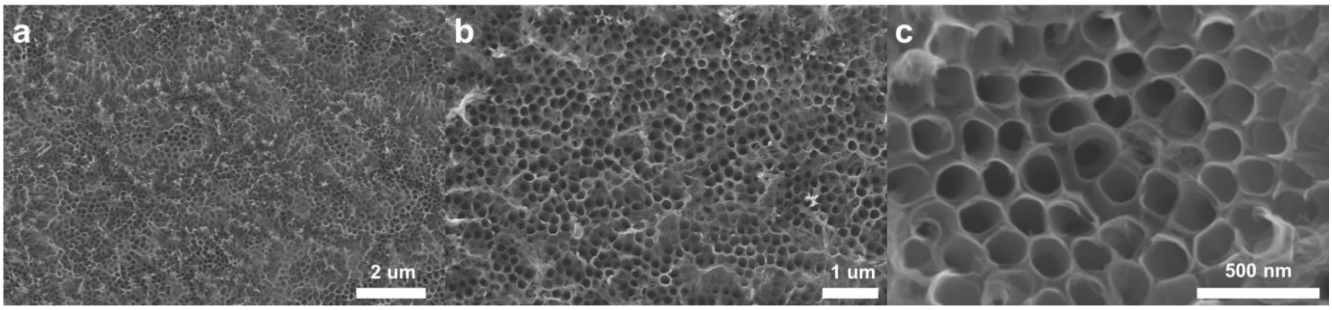

Supplementary Fig. 3. a-c SEM images of TiO_2 NTs.

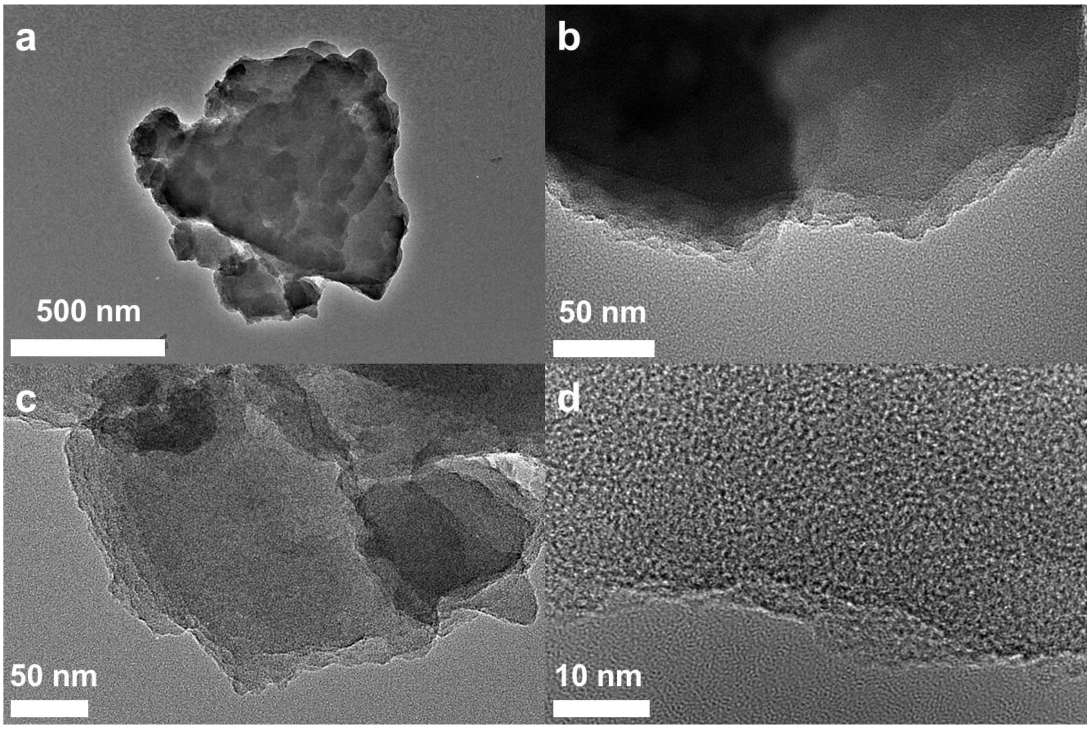

Supplementary Fig. 4. a-c TEM and **d** HRTEM images of NGA-COF.

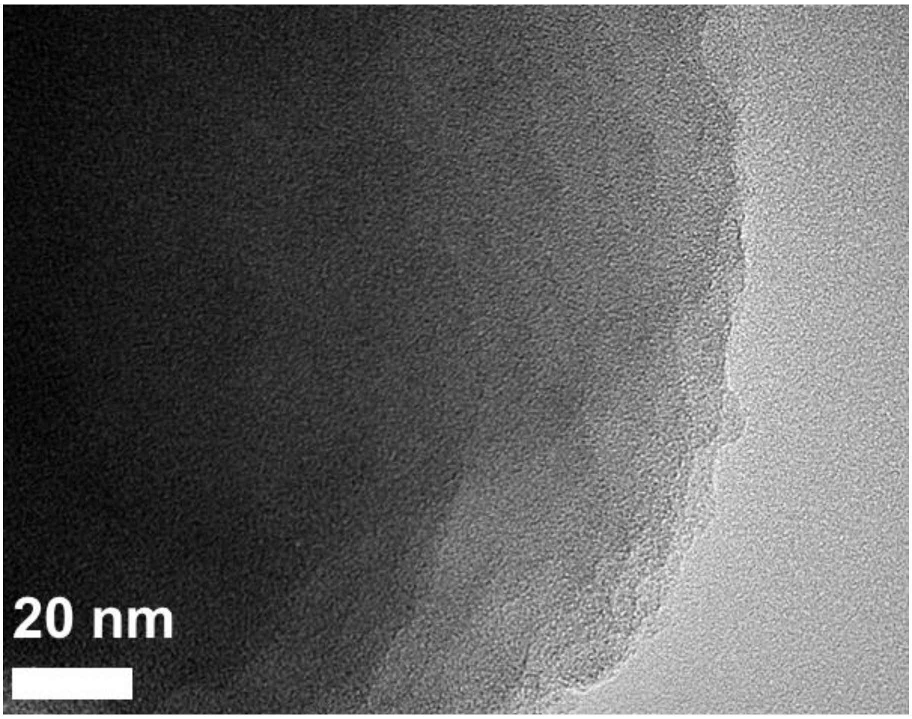

Supplementary Fig. 5. HRTEM image of the edge of NGA-COF.

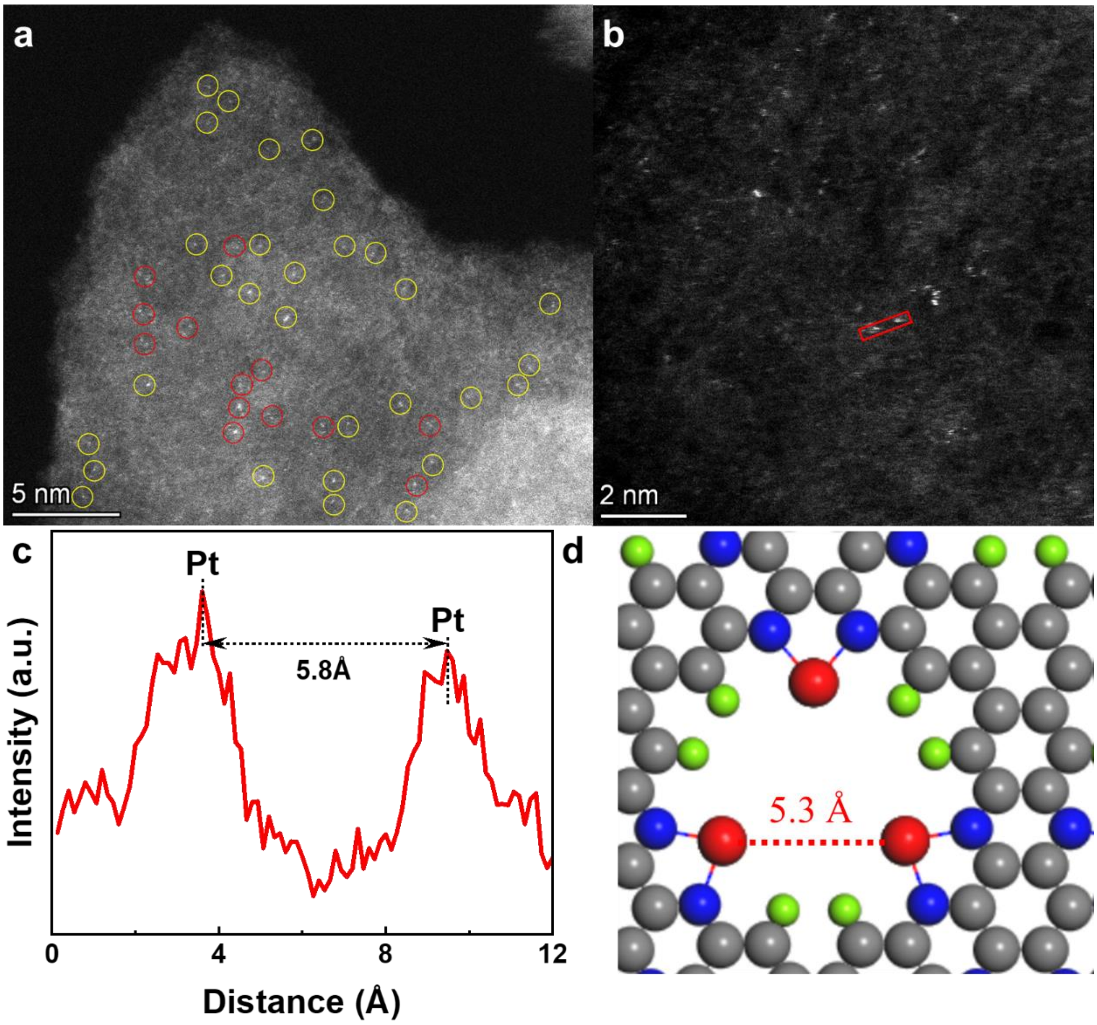

Supplementary Fig. 6. a Magnified atomic-resolution HAADF-STEM image of NGA-COF@Pt. **b** Enlarged atomic-resolution HAADF-STEM image of NGA-COF@Pt and **c** corresponding distance between two bright spots (Pt atoms) in the red box. **d** Simulated distance between two adjacent Pt atoms in NGA-COF@Pt.

Supplementary Note. Single atoms in the red circles of **Supplementary Fig. 6a** mostly appear as atomic clusters, including diatomic or triatomic forms. Distance between the two atoms is about 5.8Å as observed in atomic-resolution HAADF-STEM at larger magnifications (**Supplementary Fig. 6b-c**), which corresponds to the Pt space in the structure simulation (**Supplementary Fig. 6d**).

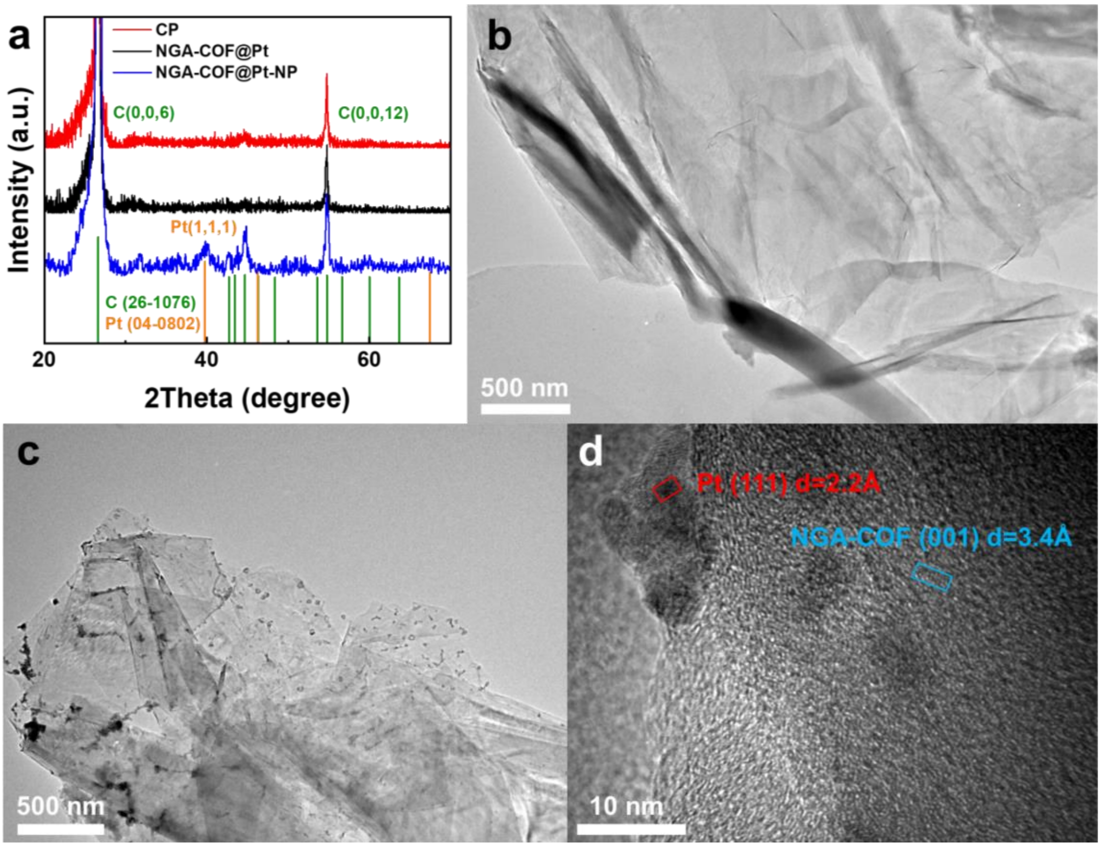

Supplementary Fig. 7. a XRD patterns of NGA-COF@Pt on CP and bare CP. TEM images of **b** Nanosheets derived from NGA-COF@Pt after electrochemical modification, **c** NGA-COF@Pt-5000 and corresponding **d** HRTEM of NGA-COF@Pt-5000.

Supplementary Note: With the segment number of CV increasing from 2000 to 2500, typical Pt (111) peak emerges in X-ray diffraction (XRD) pattern (**Supplementary Fig. 7a**). After electrochemical modification (2000 segments of CV), NGA-COF was exfoliated into nanosheets during the process[1], reducing the thickness of C-axis and the number of layers (**Supplementary Fig. 7b**), which can increase the electrochemical active surface area (this will be proved later) of the catalyst and activate the catalytic sites originally buried deep inside NGA-COF. With the segment number of CV increasing to 2500, Pt particles on NGA-COF@Pt-NP were observed by TEM (**Supplementary Fig. 7c**). Despite electrodeposition under acidic conditions (0.5 M H_2SO_4) for more than 24 h, the (001) crystal plane of NGA-COF and the layered graphene-like structure along C-axis remain. Such high stability can be attributed to the highly π–π conjugated multiring aromatic systems of NGA-COF (**Supplementary Fig. 7d**).

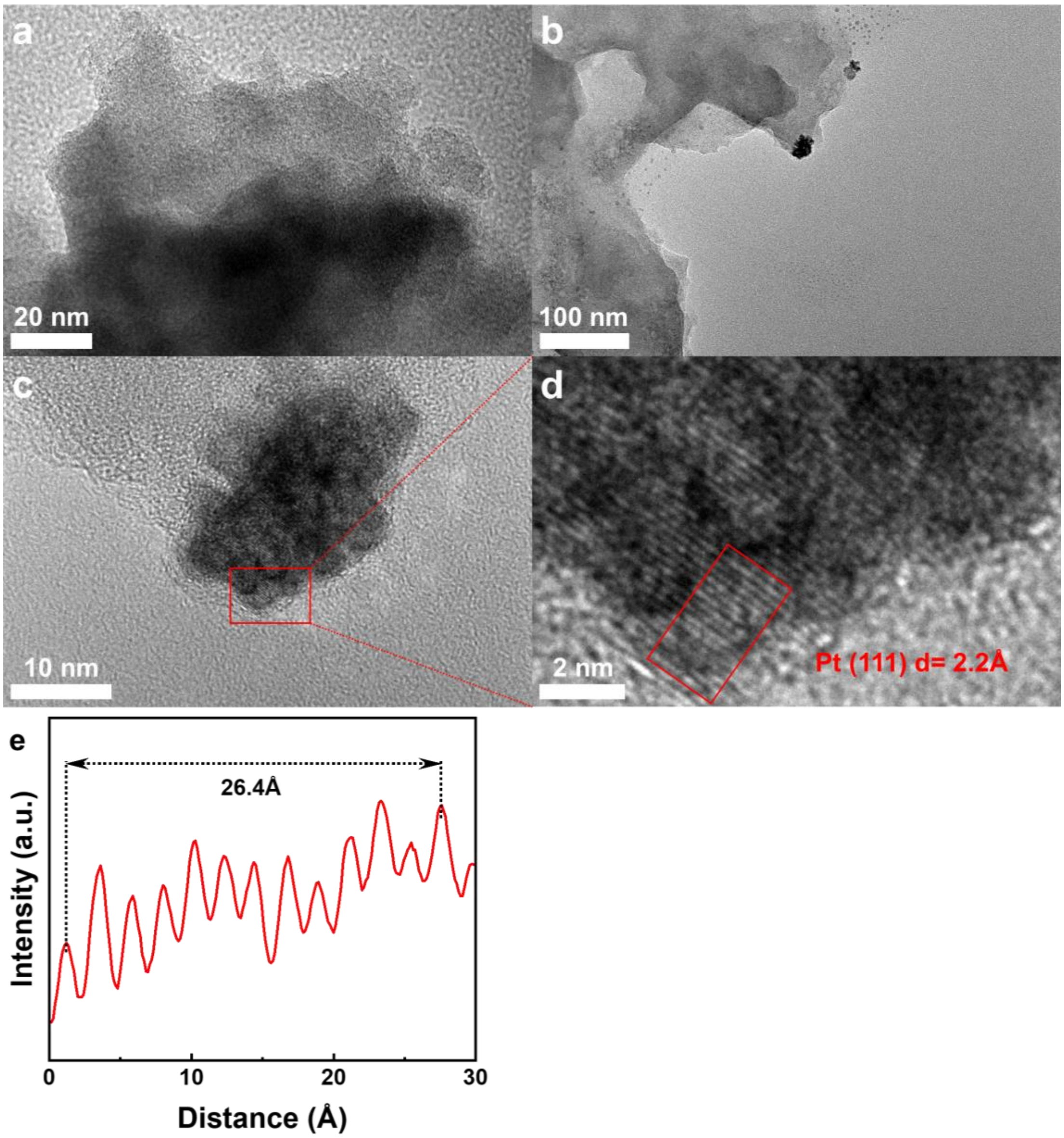

Supplementary Fig. 8. TEM images of **a** NGA-COF@Pt (2000 segments of CV) and **b** NGA-COF@Pt-NP (2500 segments of CV). **c-d** Magnified HRTEM images of NGA-COF@Pt-NP. **e** Corresponding lattice spacing in the red box of **Supplementary Fig. 8d**.

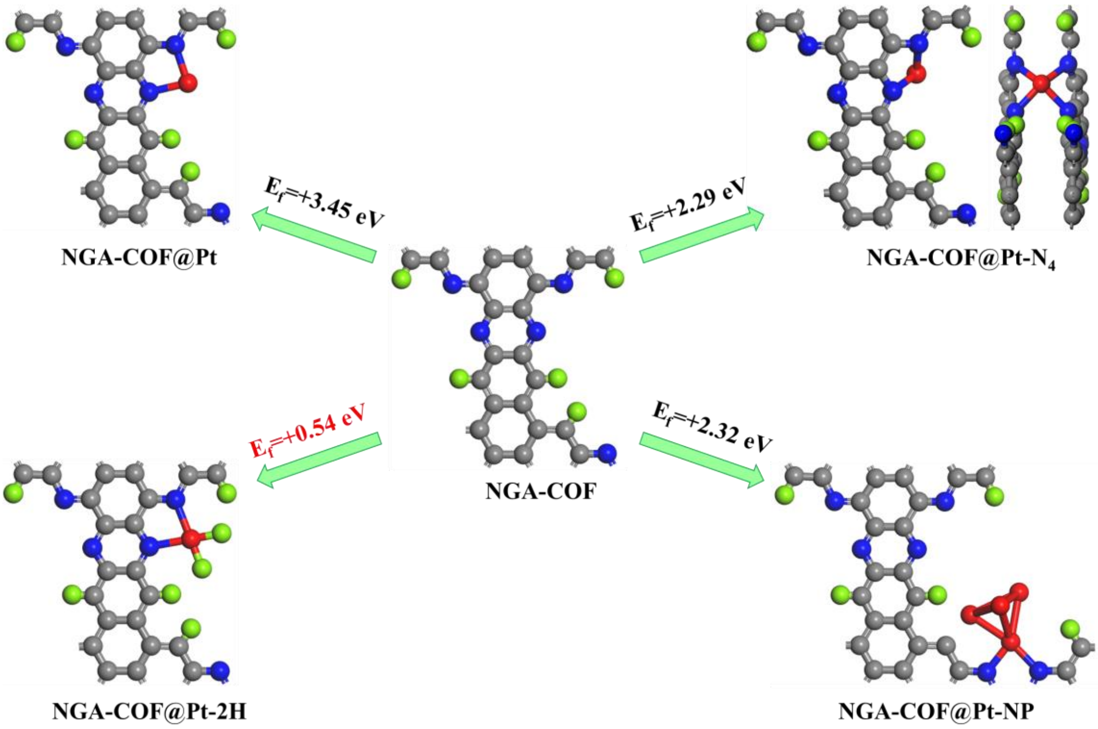

Supplementary Fig. 9. Formation paths and corresponding calculated formation energies of different samples.

Supplementary Note: Although NGA-COF@Pt-NP is more thermodynamically stable than NGA-COF@Pt theoretically, the formation energy of these two structures is too high (3.45 eV for NGA-COF@Pt and +2.32 for NGA-COF@Pt-NP) for them to form at normal temperature and pressure spontaneously. Therefore, electrochemical modification synthetic method was applied to overcome the activation energy barrier required for the forming of Pt single atoms on NGA-COF. This method can control the reaction process by adjusting specific and quantifiable parameters such as voltage, current and deposition time. Furthermore, a general electrochemical modification strategy has also been developed to prepare conductive agent-free COF-based electrocatalysts at room temperature and pressure, which will not waste a large amount of solution containing noble metal ions due to subsequent processes such as centrifugation and filtration as liquid-phase or hydrothermal synthesis[2,3]. Besides, the mild electrochemical synthesis method can also avoid the agglomeration of single atom sites due to excessive surface energy at high temperatures as in the traditional pyrolysis method[4]. Such a controllable, facile and low-cost electrochemical modification method can dramatically reduce the waste of noble metals by minimizing the usage of Pt during the whole synthesis process. During the electrochemical modification process, the proton-rich acidic environment and the applied reduction voltage on the WE facilitated the conversion of the unstable NGA-COF@Pt (E_f = +3.45 eV) intermediate to a more stable configuration, NGA-COF@Pt-2H (E_f = +0.54 eV).

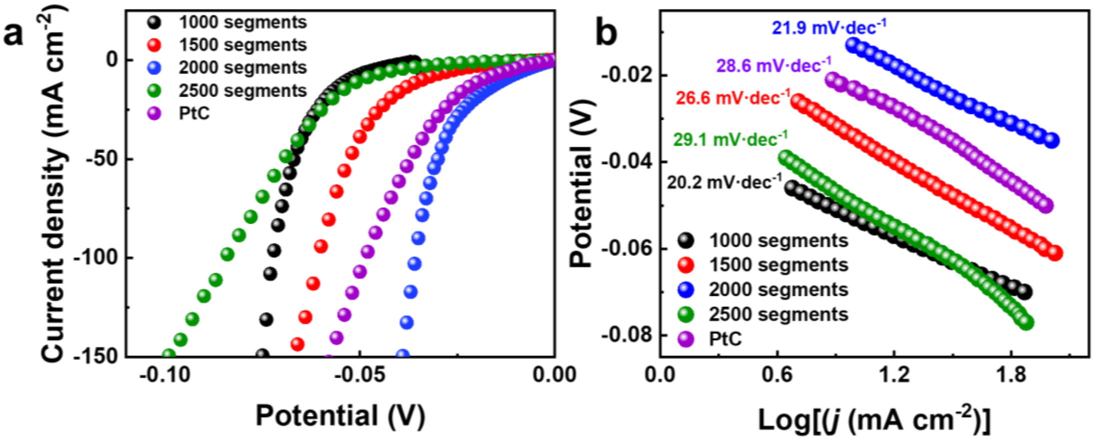

Supplementary Fig. 10. a Polarization curves and **b** corresponding Tafel plots of NGA-COF@Pt after electro-deposition for different segments.

Supplementary Note: It can be concluded that CV for 2000 segments is the best electrochemical modification parameter to form NGA-COF@Pt among all the samples tested above. When the segments are less than 2000, the occupation of Pt single atoms into NGA-COF is not adequate, whereas when the CV is more than 2000 segments, Pt particles will be formed, resulting in the decrease of active sites and the degradation of catalytic performance.

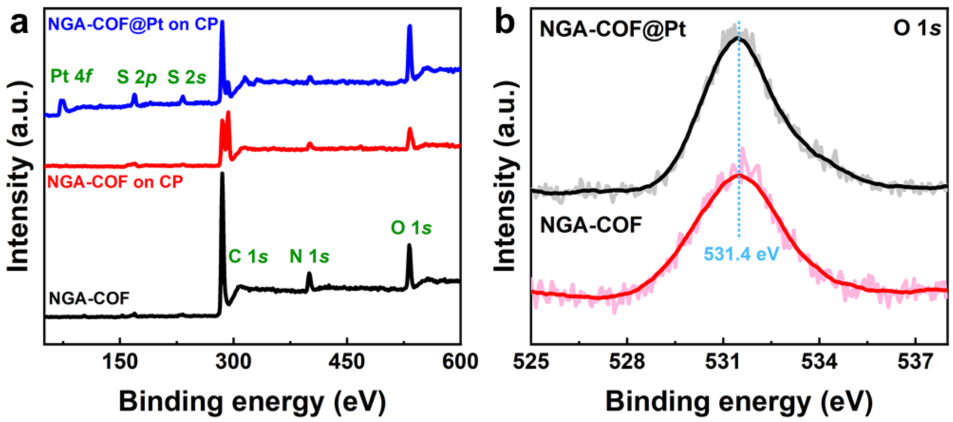

Supplementary Fig. 11. a XPS survey spectra of different samples. **b** High-resolution XPS spectra of O 1*s* of NGA-COF and NGA-COF@Pt.

Supplementary Note: The full spectrum in **Supplementary Fig. 11a** confirms the introduction of Pt after electrochemical modification. High-resolution XPS spectra of O 1*s* (**Supplementary Fig. 11b**) demonstrate that the chemical environments of O remained the same after the introduction of Pt, indicating that Pt was not directly coordinated with O.

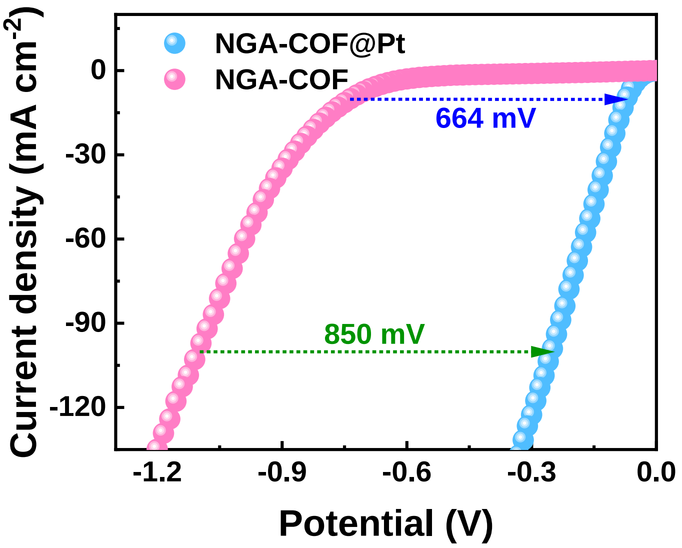

Supplementary Fig. 12. Polarization curves of NGA-COF and NGA-COF@Pt without iR-compensation in 0.5 M H_2SO_4.

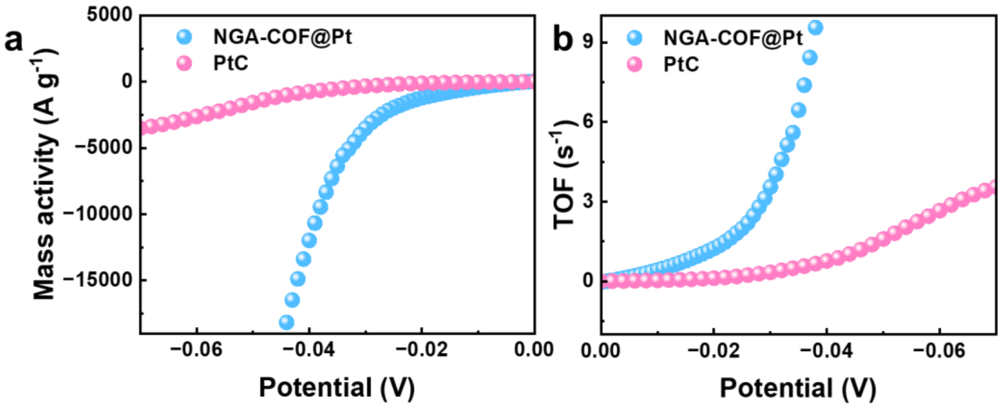

Supplementary Fig. 13. a Mass activity and **b** TOF plotted as a function of the potential for NGA-COF@Pt and PtC in 0.5 M H_2SO_4.

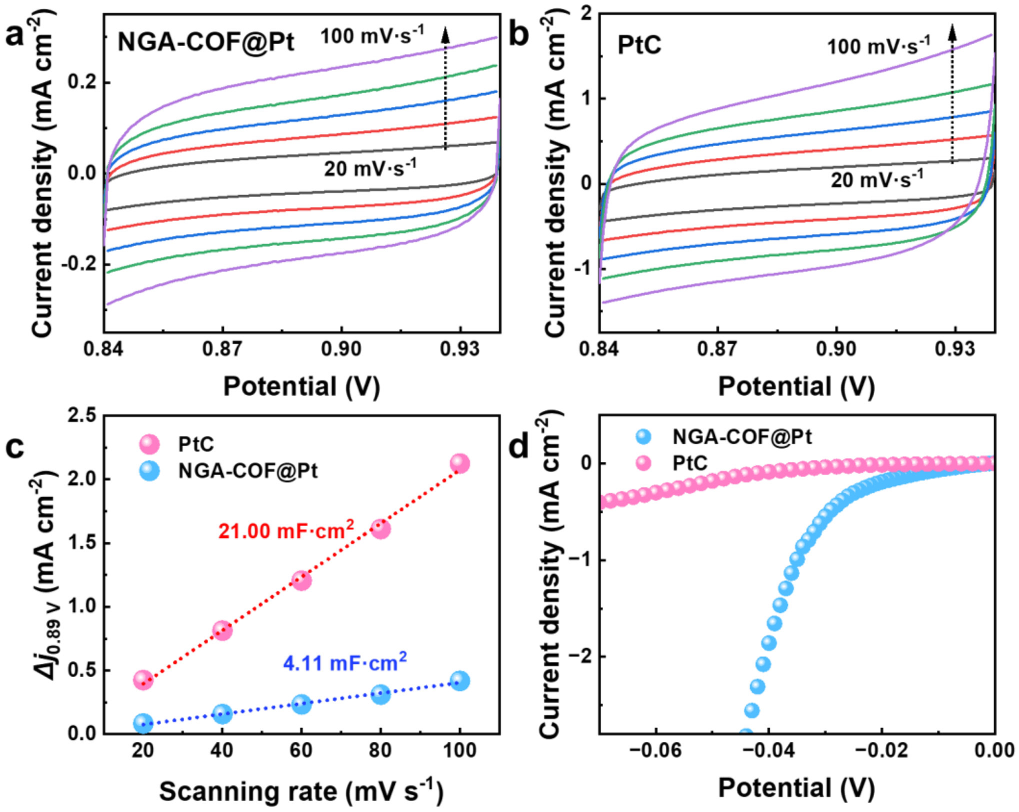

Supplementary Fig. 14. CV curves of **a** NGA-COF@Pt and **b** PtC in different scanning rates. **c** C_{dl} calculated with Δj at 0.89 V. **d** Polarization curves normalized by ECSA. Electrolyte: 0.5 M H_2SO_4.

Supplementary Note: ECSA is estimated by CVs with ECSA = C_{dl}/C_s (C_{dl} is double layer capacitance, C_s is capacitive behavior)[5]. The specific capacitance can be converted into ECSA using an average C_s value of 40 μF cm^{-2} according to literature[6]. Therefore, ECSA of PtC and NGA-COF@Pt can be calculated to be 525.0 and 102.8 cm^2 in 0.5 M H_2SO_4.

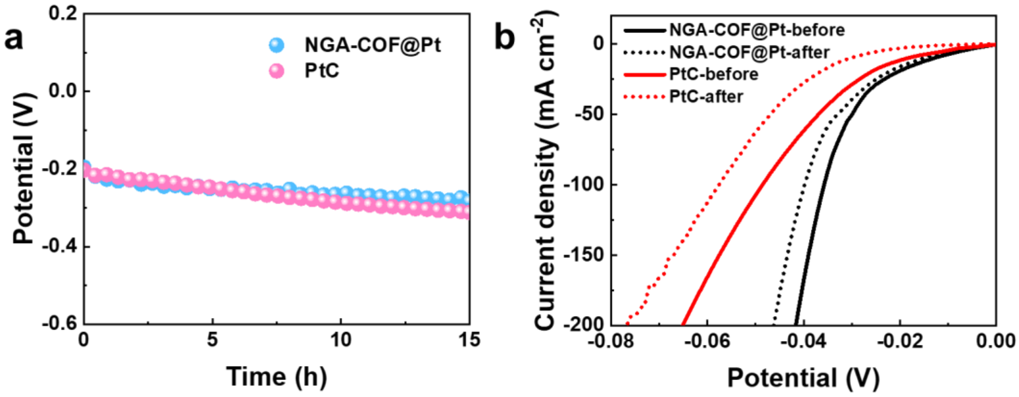

Supplementary Fig. 15. a Chronopotentiometric tests and **b** corresponding polarization curves of different samples before and after chronopotentiometric tests in 0.5 M H_2SO_4.

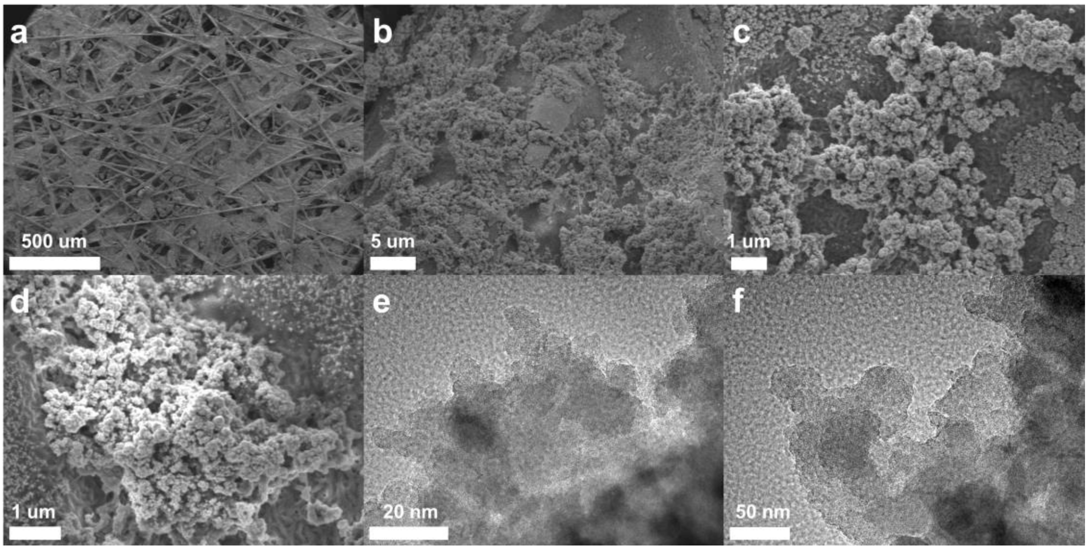

Supplementary Fig. 16. a-d SEM images and **e-f** TEM images at different magnifications of NGA-COF@Pt after chronopotentiometric test in 0.5 M H_2SO_4.

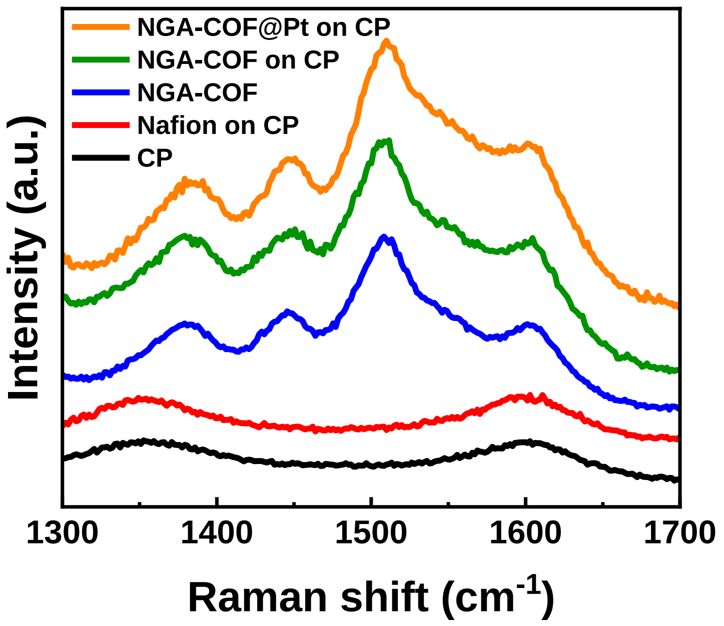

Supplementary Fig. 17. Ex-situ Raman spectra of different samples prior to electrochemical tests.

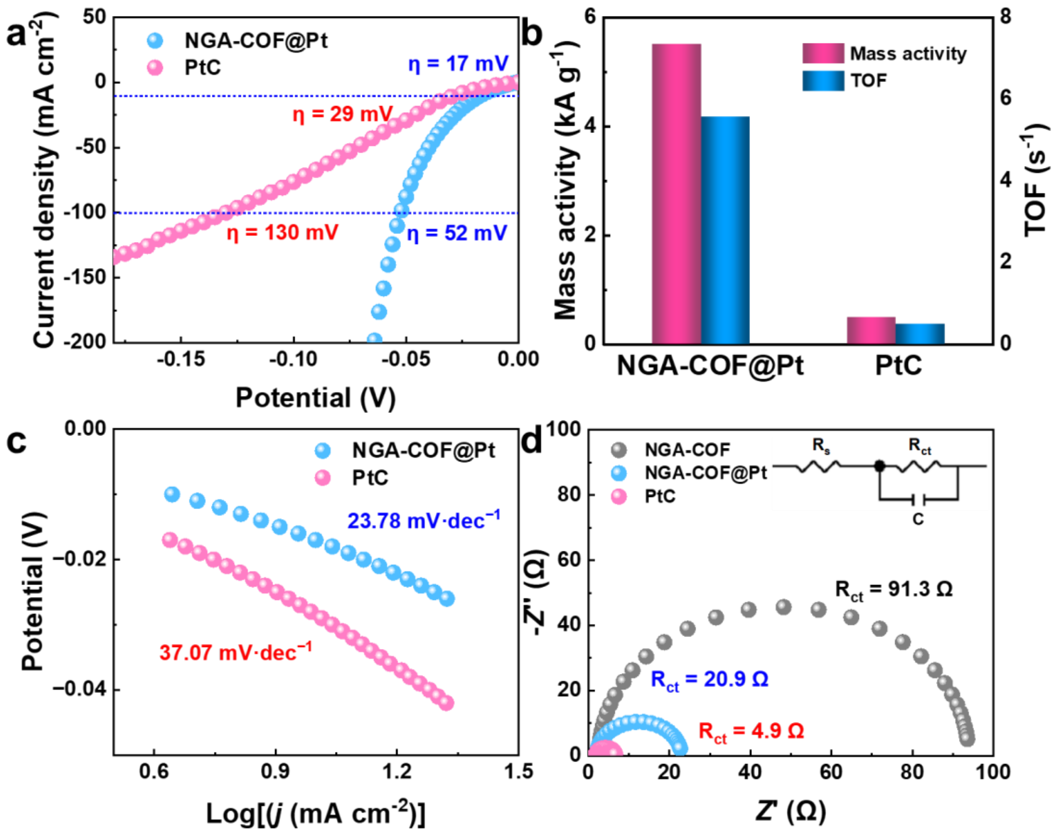

Supplementary Fig. 18. **a** Polarization curves, **b** mass activity and TOF, **c** corresponding Tafel plots and **d** EIS of different samples (inset shows the equivalent circuit diagram) in 1 M KOH.

Supplementary Note: NGA-COF@Pt exhibits lower overpotential (17 mV) at 10 mA cm^{-2} than PtC (**Supplementary Figs. 18a and 19**). It also demonstrates outstanding mass activity and TOF (5502 A g^{-1} and 5.56 s^{-1}) at overpotential of 50 mV, more than ten times than that of PtC (486 A g^{-1} and 0.49 s^{-1}), surpassing that of noble metal materials reported recently (**Supplementary Figs. 18b, 20 and Table 4**), too. Besides, NGA-COF@Pt also possesses lower Tafel slop (23.78 mV dec^{-1}, **Supplementary Fig. 18c**), smaller electron transfer resistance (20.9 Ω, **Supplementary Fig. 18d and Supplementary Table 2**), higher ECSA normalized current density (**Supplementary Fig. 21**) and better stability (**Supplementary Fig. 22**). Interestingly, after the stability test, the particle size of NGA-COF@Pt under alkaline conditions still remained almost original (**Supplementary Fig. 23**) while it reduced under acidic conditions (**Supplementary Fig. 16**). It is possible that imine bonds are more stable in alkaline than acidic conditions[7], resulting in the decrease of the polymerization degree of NGA-COF@Pt under acidic conditions.

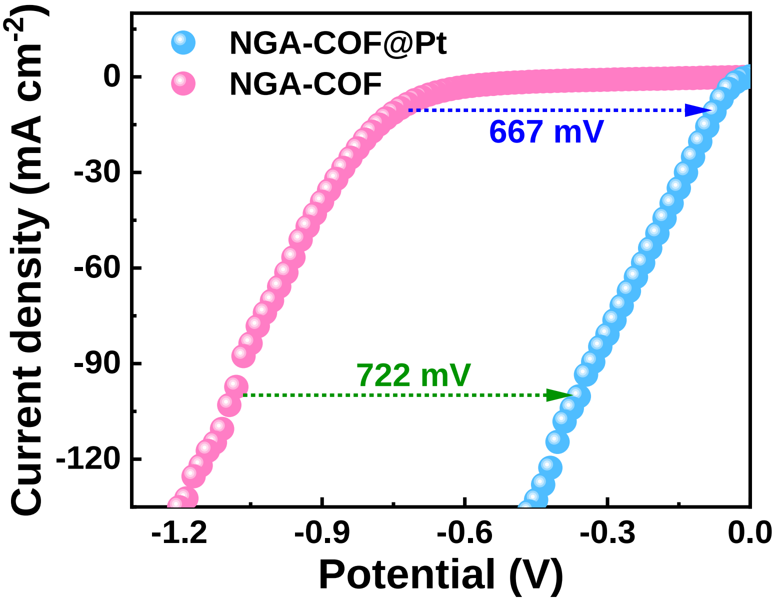

Supplementary Fig. 19. Polarization curves of NGA-COF and NGA-COF@Pt without iR-compensation in 1 M KOH.

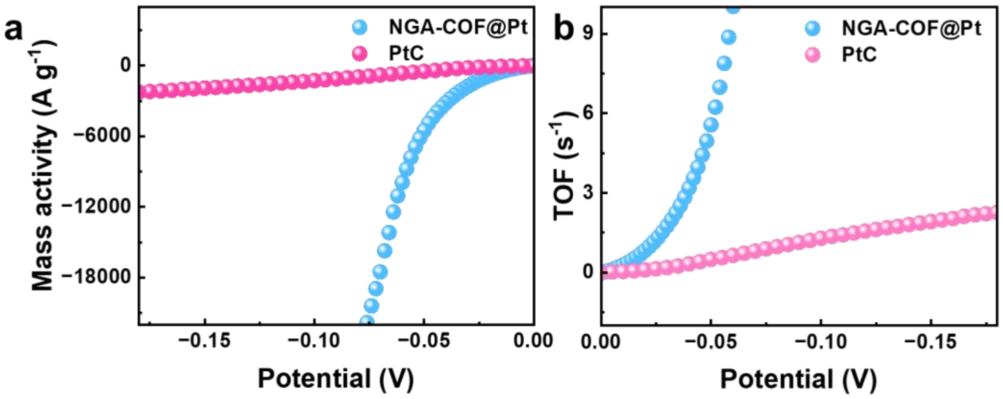

Supplementary Fig. 20. **a** Mass activity and **b** TOF plotted as a function of potential for NGA-COF@Pt and PtC in 1 M KOH.

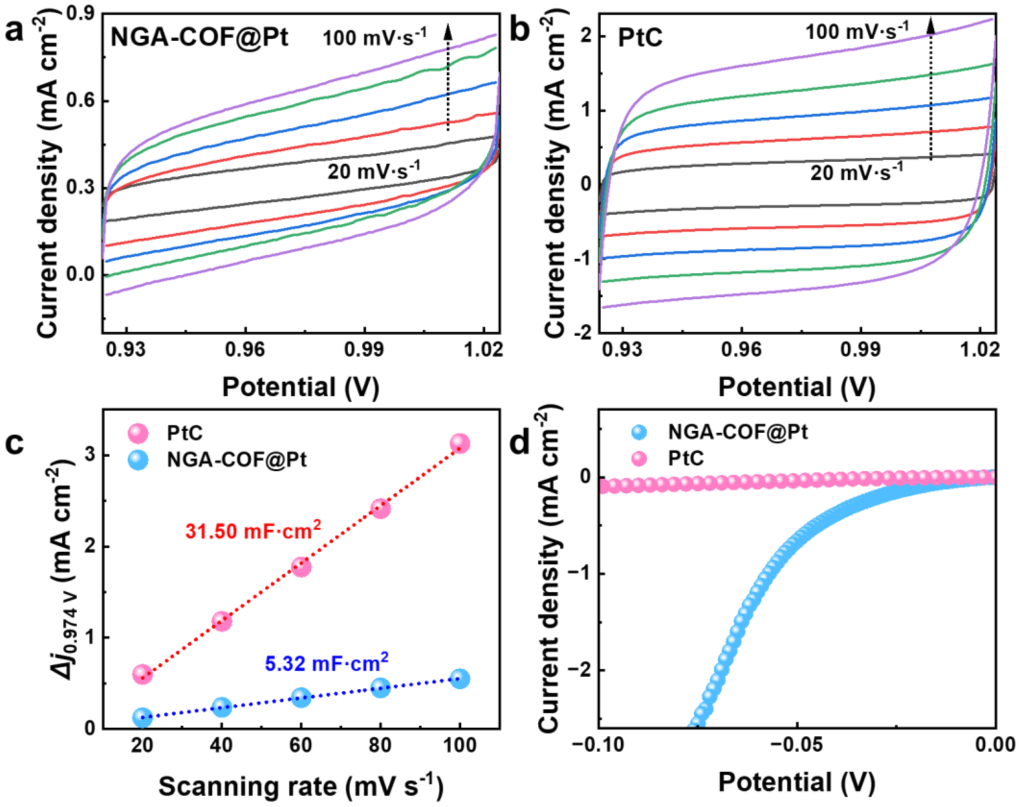

Supplementary Fig. 21. CV curves of **a** NGA-COF@Pt and **b** PtC in different scanning rates. **c** C_{dl} calculated with ΔJ at 0.974 V. **d** Polarization curves normalized by ECSA. Electrolyte: 1 M KOH.

Supplementary Note. The ECSA of PtC and NGA-COF@Pt is calculated to be 787.5 and 133.0 cm^2 in 1 M KOH.

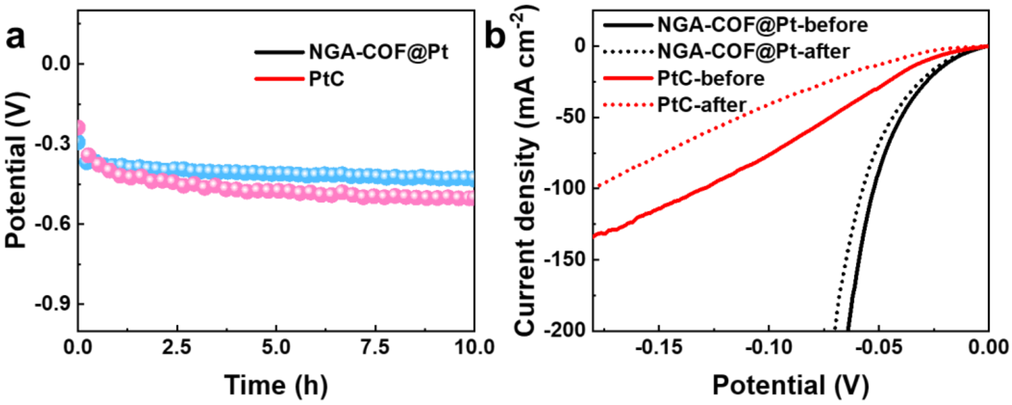

Supplementary Fig. 22. a Chronopotentiometric tests and **b** corresponding polarization curves of different samples prior to and after chronopotentiometric tests in 1 M KOH.

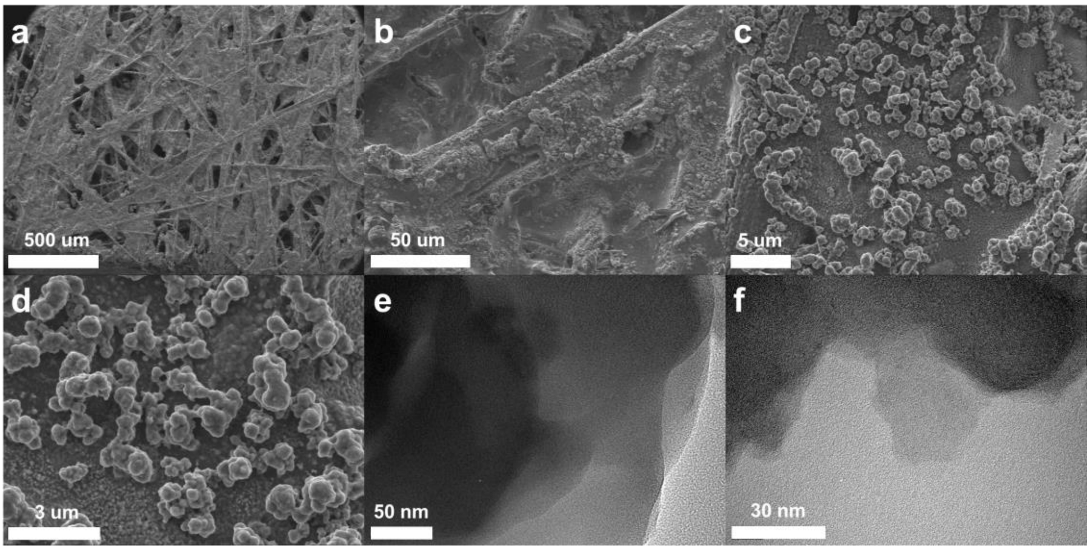

Supplementary Fig. 23. a-d SEM images and **e-f** TEM images at different magnifications of NGA-COF@Pt after chronopotentiometric tests in 1 M KOH.

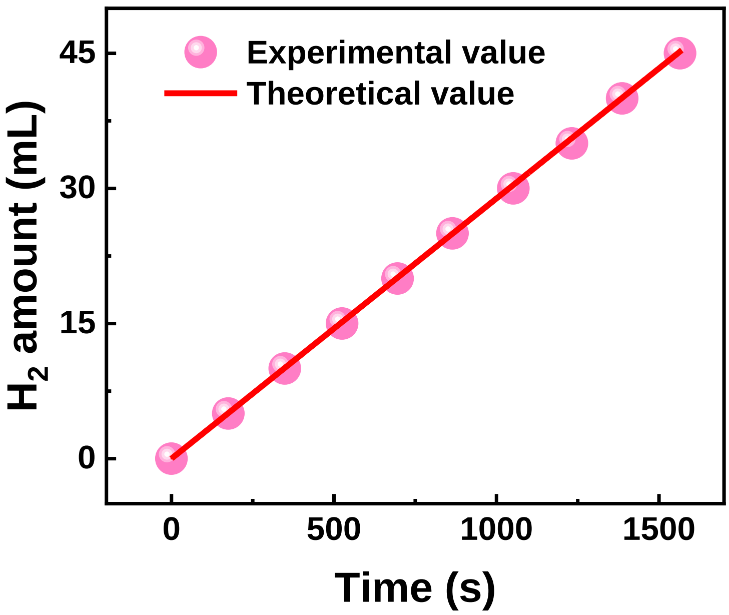

Supplementary Fig. 24. Experimental and theoretical value of H_2 production by using NGA-COF@Pt as working electrode. (FE = 99.53%).

Supplementary Note: Catalytic efficiency was tested by a home-made drainage device and a Faraday efficiency (FE) of 99.53 % was obtained.

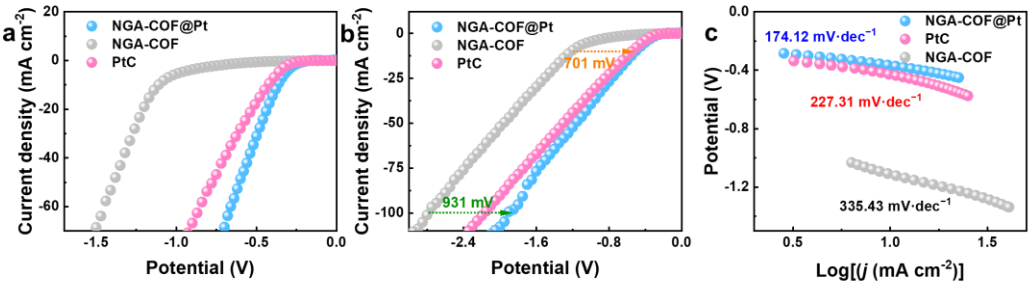

Supplementary Fig. 25. Polarization curves **a** with or **b** without iR-compensation and **c** corresponding Tafel plots of NGA-COF, NGA-COF@Pt and PtC in 0.1 M PBS (pH 7.4).

Supplementary Note: In addition, the electrochemical performance of NGA-COF@Pt towards HER in a neutral environment (0.1 M PBS, pH 7.4) was also recorded. LSV curves were collected in 0.1 M PBS (pH 7.4) solution with saturated calomel electrode (SCE) used as RE ($E_{RHE} = E_{SCE} + 0.241 + 0.059$ pH). Although the overpotential needed for NGA-COF@Pt (368 mV) to achieve 10 mA cm^{-2} is lower than that of commercial PtC (429 mV), this value is still far from practical applications. Therefore, there is room for the improvement and optimization of the catalytic performance of NGA-COF@Pt towards HER under neutral conditions.

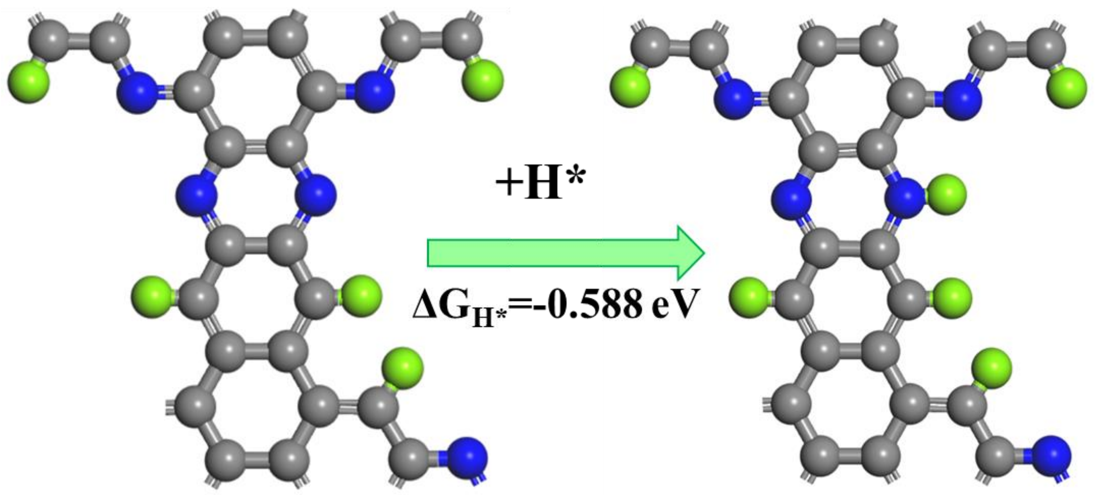

Supplementary Fig. 26. Configurations of H* intermediate adsorbed on the N site of NGA-COF.

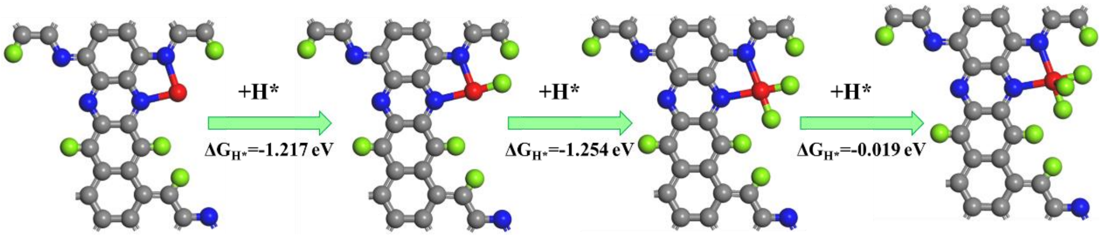

Supplementary Fig. 27. Configurations of H* intermediate adsorbed on the Pt site of NGA-COF@Pt, NGA-COF@Pt-H and NGA-COF@Pt-2H, respectively.

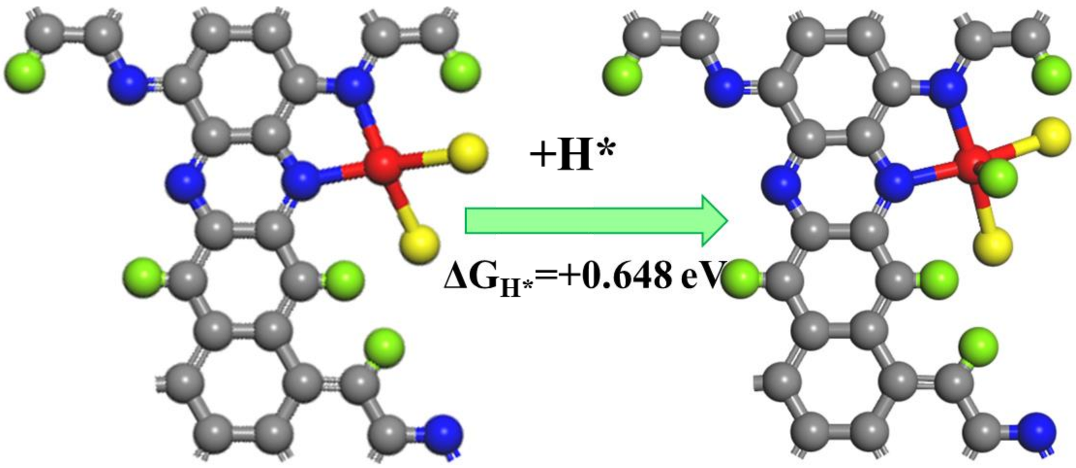

Supplementary Fig. 28. Configurations of H* intermediate adsorbed on the Pt site of NGA-COF@Pt-2Cl.

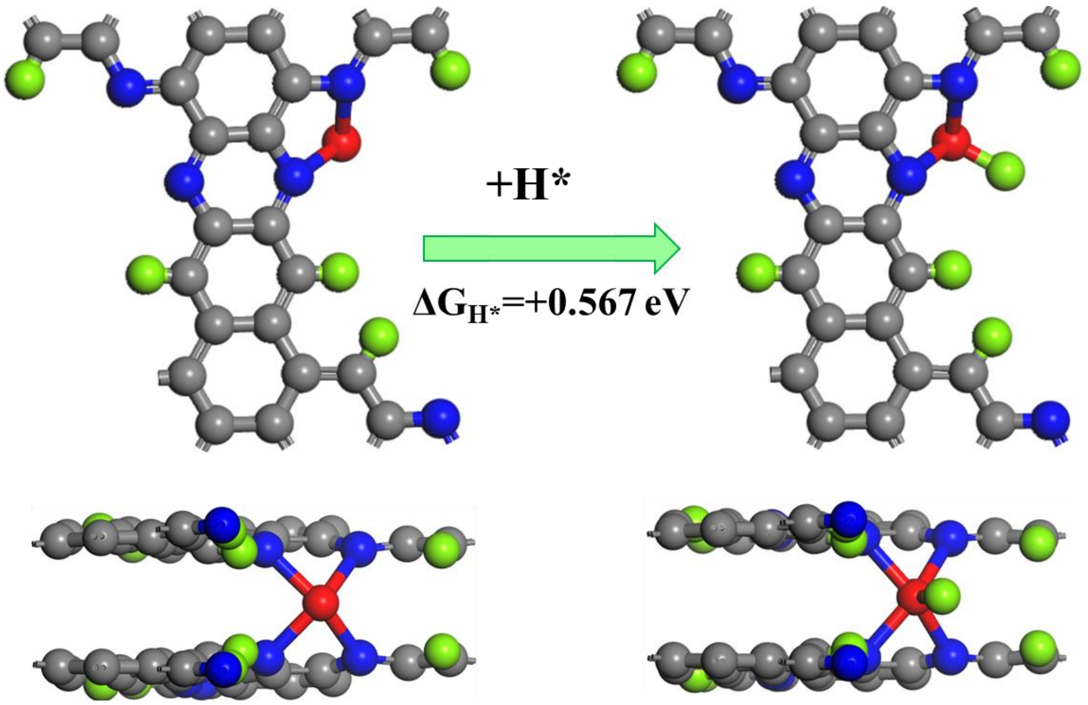

Supplementary Fig. 29. Configurations of H* intermediate adsorbed on the Pt site of NGA-COF@Pt-N_4 alone C (the top two pictures) and B axis (the bottom two pictures).

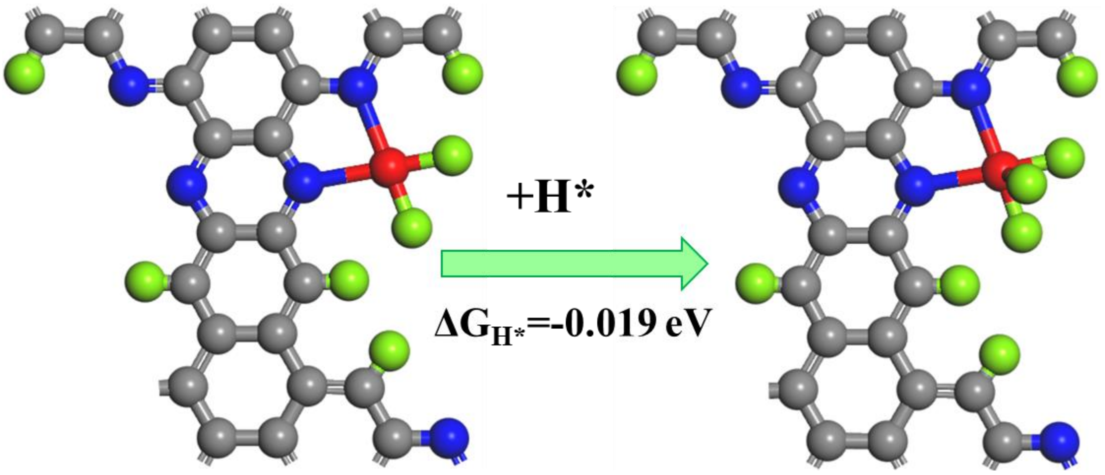

Supplementary Fig. 30. Configurations of H* intermediate adsorbed on the Pt site of NGA-COF@Pt-2H.

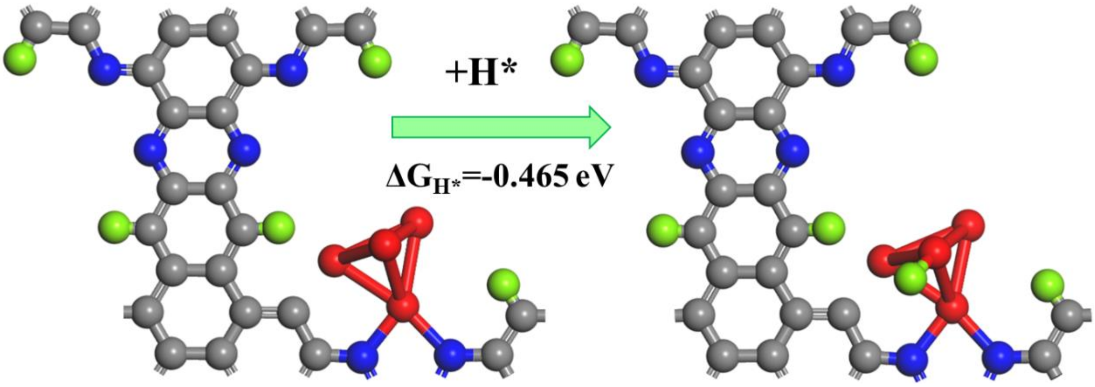

Supplementary Fig. 31. Configurations of H* intermediate adsorbed on the Pt site of NGA-COF@Pt-NP.

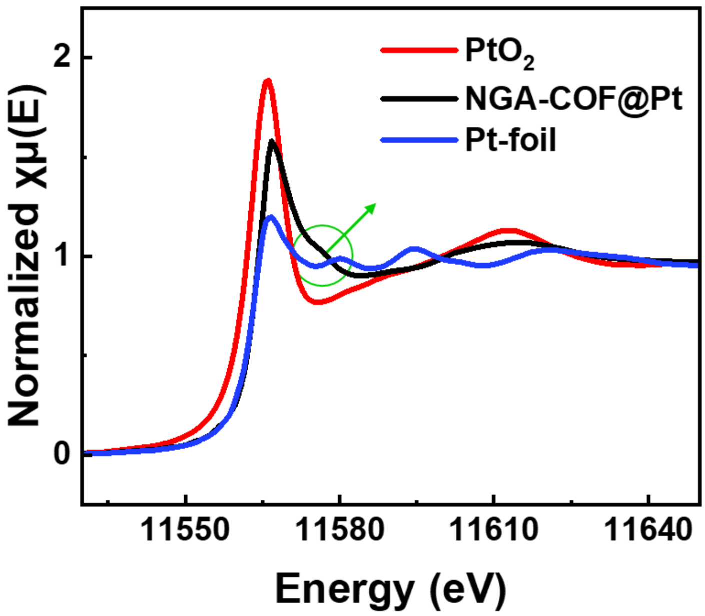

Supplementary Fig. 32. Pt L_3-edge XANES spectra of Pt-foil, NGA-COF@Pt and PtO_2.

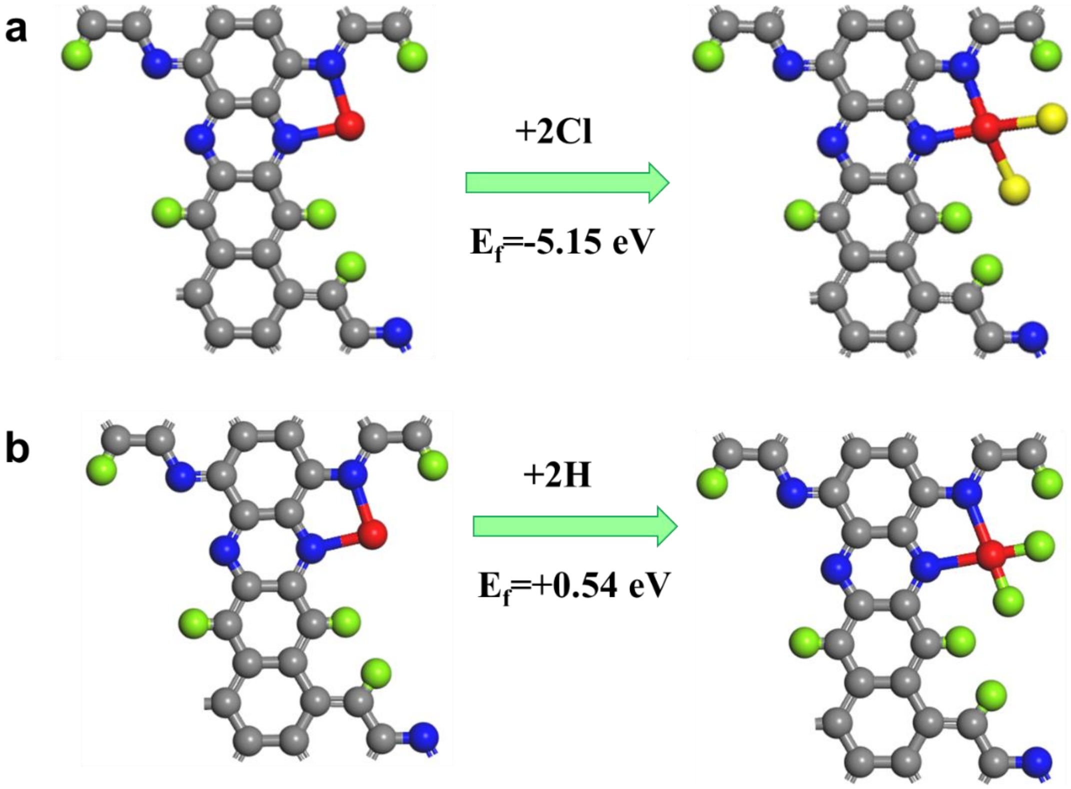

Supplementary Fig. 33. Reaction path and formation energy of **a** NGA-COF@Pt to NGA-COF@Pt-2Cl and **b** NGA-COF@Pt to NGA-COF@Pt-2H.

Supplementary Note: Theoretically, NGA-COF@Pt-2Cl is easier to form than NGA-COF@Pt-2H from NGA-COF@Pt. Therefore, if the real configuration of the catalyst before the Zeta potential test is NGA-COF@Pt, it should form NGA-COF@Pt-2Cl after the test (in 1 mmol L^{-1} KCl solution).

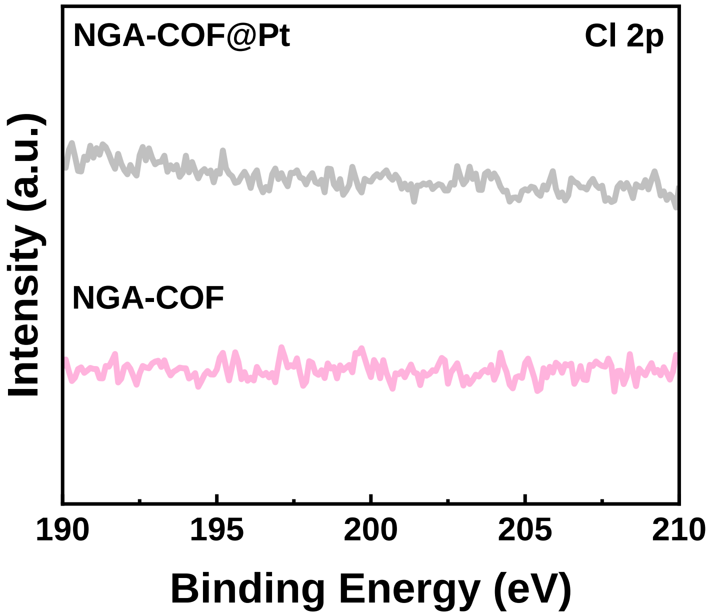

Supplementary Fig. 34. High-resolution XPS spectra of Cl 2p of NGA-COF and NGA-COF@Pt after Zeta potential test.

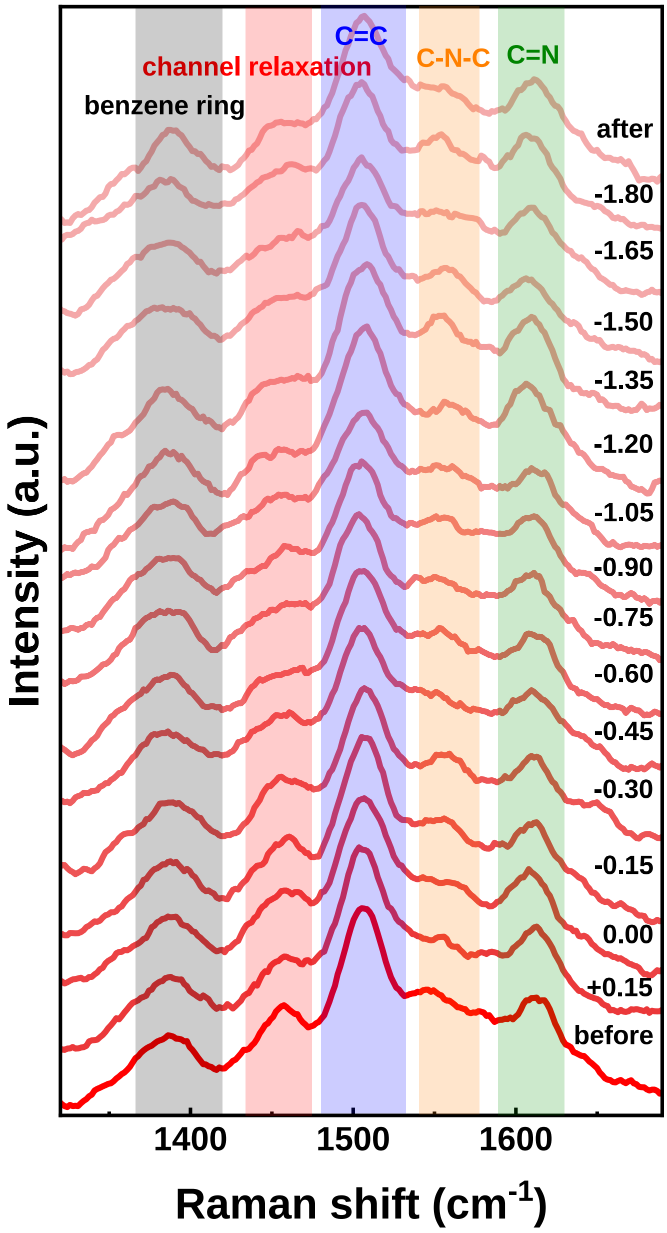

Supplementary Fig. 35. In situ Raman analysis of NGA-COF@Pt during the HER process at different potentials in 1 M KCl.

Supplementary Note: Chloride ions and potassium ions may affect the respiration effect of COF by filling the micropores of NGA-COF in the HER process. Therefore, some intensity fluctuations may occur in the in-situ Raman spectra including channel relaxation at 1462 cm^{-1} and C-N-C bridge vibration at 1559 cm^{-1}. These fluctuations were not observed in the previous 0.5 M sulfuric acid (**Fig. 5e**) because the hydrogen ion radius was too small and the sulfate ion radius was too large to fill into the micropores of NGA-COF during the HER process.

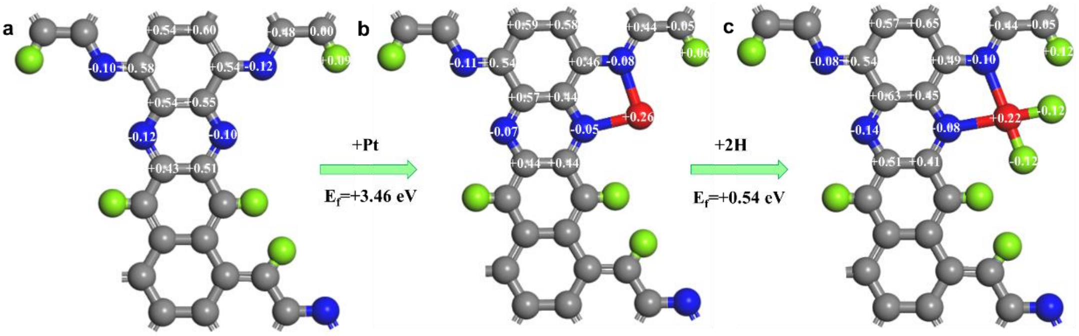

Supplementary Fig. 36. Configurations of **a** NGA-COF, **b** NGA-COF@Pt and **c** NGA-COF@Pt-2H with Bader charge analysis marked on specific atoms, respectively, and the corresponding formation energy between these configurations.

Supplementary Note: According to Bader charge analysis, in NGA-COF@Pt-2H, the two H atoms connected to Pt are negatively charged, which can stabilize the excess positive charge on Pt. These two negative H atoms can neutralize the excess positive charge on Pt and regulate the electronic structure of Pt. Since there is only H^+ in the electrochemically modified solution (0.5 M H_2SO_4), the two negative H atoms can only be realized at the electrochemical cathode potential applied. This also explains why the formation energy is higher than 0 eV. In addition, after two H atoms are attached, the electronic structure of N atoms and benzene ring near Pt (**Supplementary Fig. 36c**) is closer to the original NGA-COF (**Supplementary Fig. 36a**), which reduces the risk of structural distortion of COF caused by the introduction of Pt (**Supplementary Fig. 36b**) and increases the stability of the two-dimensional structure of COF during the HER process (corroborated with the in-situ Raman spectra results).

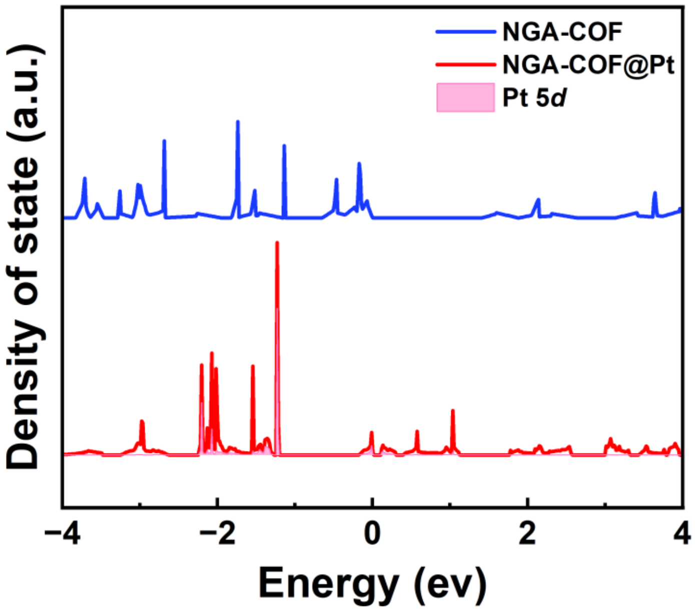

Supplementary Fig. 37. Calculated TDOS of NGA-COF and NGA-COF@Pt.

Supplementary Note: The narrowing of the band gap after doping of Pt single atoms is mainly due to the contribution of Pt 5*d* orbitals to the electronic states near the Fermi level.

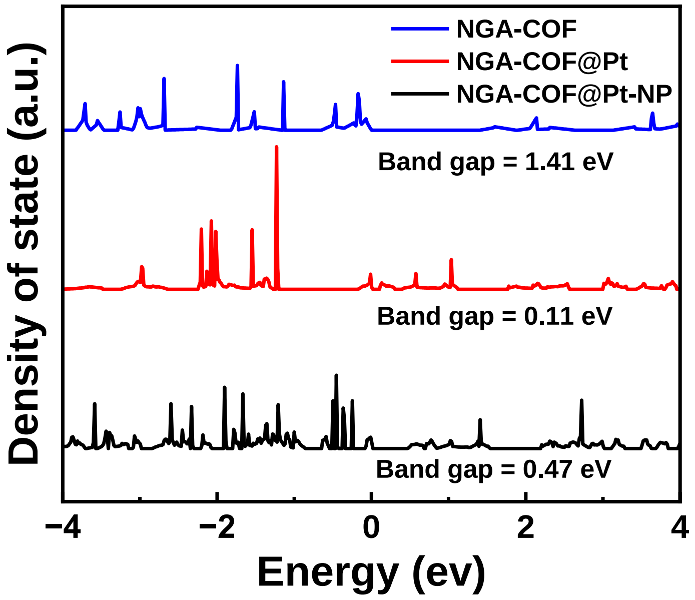

Supplementary Fig. 38. Calculated TDOS of NGA-COF, NGA-COF@Pt and NGA-COF@Pt-NP.

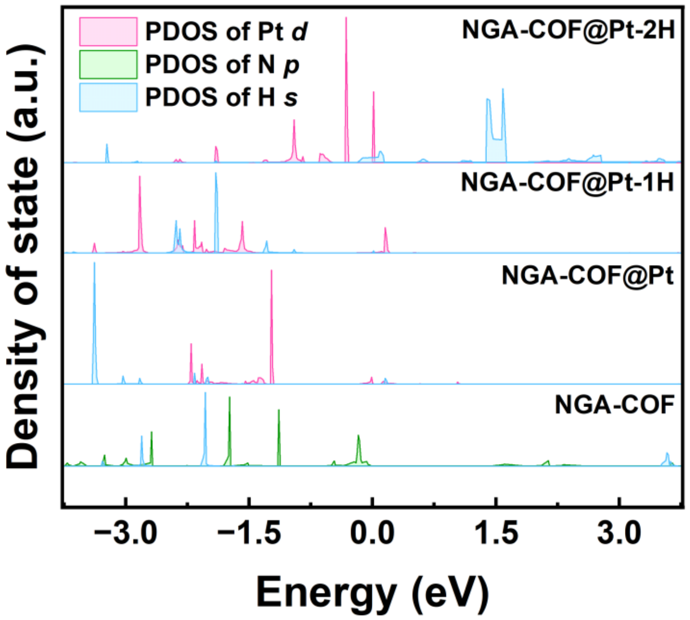

Supplementary Fig. 39. Calculated PDOS of Pt *d* orbital (NGA-COF@Pt, NGA-COF@Pt-H and NGA-COF@Pt-2H), N *p* orbital (NGA-COF) and corresponding *s* orbital of H* intermediate interacting with them.

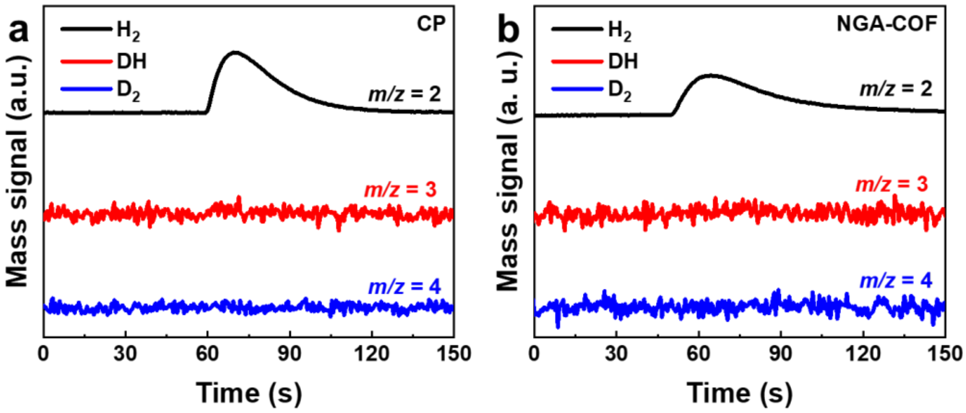

Supplementary Fig. 40. DEMS measurements of H_2, DH and D_2 signals from the reaction products for D-labeled **a** CP and **b** NGA-COF.

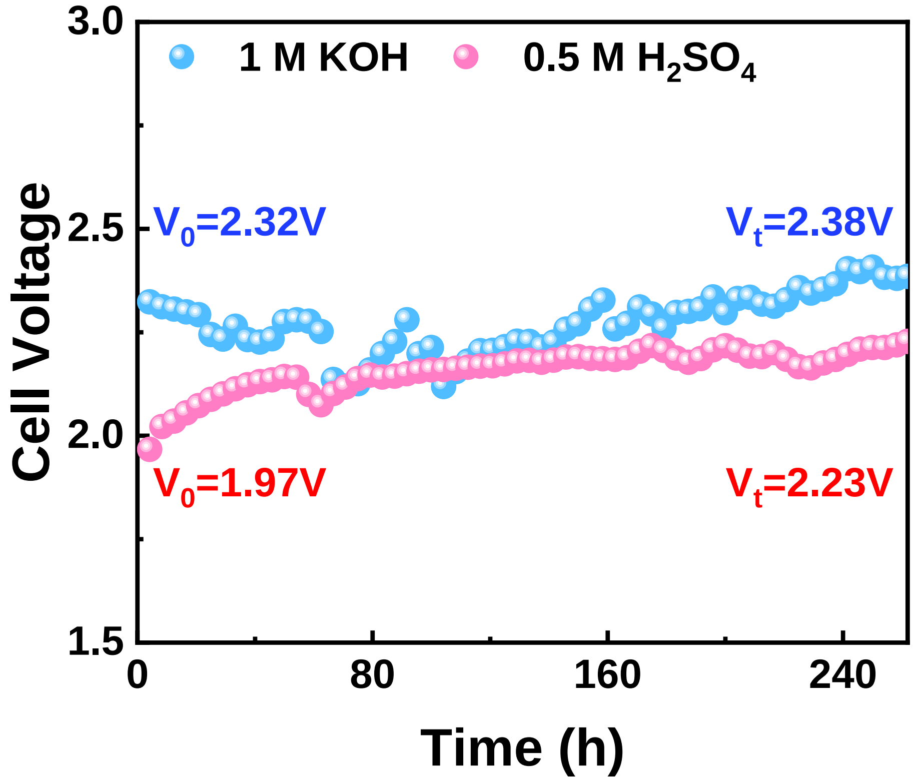

Supplementary Fig. 41. Stability tests of NGA-COF@Pt-2H||RuTiIr electrode pair towards EWS in 0.5 M H_2SO_4 or 1 M KOH under simulating working condition (constant current charging, current density = 100 mA cm^2).

Supplementary Table 1. Structural parameters obtained from the Pt L_3-edge EXAFS fitting for NGA-COF@Pt.

Path	CN	R(Å)	ΔE_0(eV)	σ^2(10^{-3} Å^2)	R factor
Pt-N	2	1.97±0.012	4.2±0.018	6.5±3.5	0.0147

Note for Table S1: CN is the coordination number for Pt-N path; R is interatomic distance; σ^2 is Debye-Waller factor (a measure of thermal and static disorder in absorber-scatterer distances); ΔE_0 is edge-energy shift (the difference between the zero kinetic energy value of the sample and that of the theoretical model) and R factor is degree of the fitting (The smaller the R value, the closer the fitting result is to the reality)[8].

Supplementary Table 2. Entire equivalent circuit fits of different samples in acidic and alkaline electrolyte.

Samples	R_s (Ω)	R_{ct} (Ω)	Electrolytes
NGA-COF	0.18	60.5	0.5 M H_2SO_4
NGA-COF@Pt	1.62	20.1	0.5 M H_2SO_4
PtC	0.65	1.3	0.5 M H_2SO_4
NGA-COF	2.6	91.3	1 M KOH
NGA-COF@Pt	2.0	20.9	1 M KOH
PtC	1.8	4.9	1 M KOH

Supplementary Table 3. Structures used for theoretical calculation in this paper and corresponding abbreviations. Atomic coordinates of the optimized computational models have been provided in the Supplementary Data 1.

	Abbreviation	Structure representation
**1**	**NGA-COF**	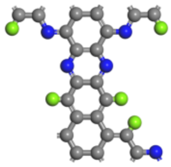
**2**	**NGA-COF@Pt**	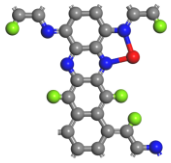
**3**	**NGA-COF@Pt-H**	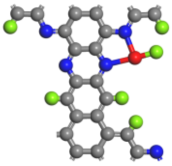
**4**	**NGA-COF@Pt-2H**	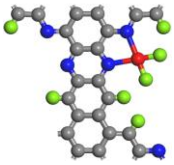
**5**	**NGA-COF@Pt-3H**	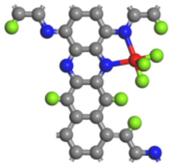
**6**	**NGA-COF@Pt-NP**	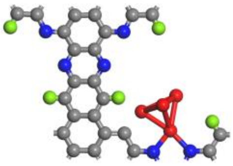
**7**	**NGA-COF@Pt-2Cl**	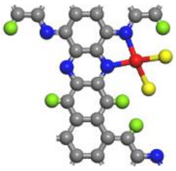
**8**	**NGA-COF@Pt-N$_4$**	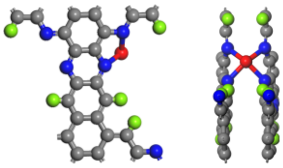

Supplementary Table 4. Comparison of noble materials electrocatalytic HER performances in acidic electrolyte.

Electrocatalysts	η_{10} (mV)	η_{100} (mV)	Tafel slop (mV dec^{-1})	Mass activity (A g^{-1})	TOF (s^{-1})	Ref.
Pt_1/N-C	19	36	14.2	N/A	22.07~η_{50}	9
IrCo@N-C	24	100	23	N/A	N/A	10
$Mo_2TiC_2T_x$-Pt_{SA}	30	77	30	8300~η_{77}	N/A	1
Pt_{SA}/OLC	38	100~η_{55}	36	7400~η_{38}	40.78~η_{100}	11
PtN_x/TiO_2	67	N/A	34	37500~η_{50}	37.9~η_{50}	12
NM-HEA NPs	60	N/A	N/A	N/A	3.1~η_{50}	13
Ru@Ni-MOF	37	112	33	N/A	N/A	14
Ni@NiP-Ru	51	95~η_{65}	35	N/A	1.1~η_{100}	15
Pt_{SA}/m-WO_{3-x}	38	N/A	45	12800~η_{50}	35~η_{100}	16
C_3N_4–Ru	140	N/A	57	N/A	N/A	17
Pt SASs/AG	12	N/A	29.3	22400~η_{50}	N/A	18
Pt@DG	30	N/A	53	26050~η_{100}	26410~η_{100}	19
**Pt/C**	**23**	**51**	**29**	**1013~η_{44}**	**1.02~η_{44}**	**This**
**NGA-COF@Pt**	**13**	**35**	**22**	**18165~η_{44}**	**18.4~η_{44}**	**work**

Supplementary Table 5. Comparison of noble materials electrocatalytic HER performances in alkaline electrolyte.

Electrocatalysts	η_{10} (mV)	η_{100} (mV)	Tafel slop (mV dec^{-1})	Mass activity (A g^{-1})	TOF (s^{-1})	Ref.
Pt@DG	37	N/A	69	6780~η_{100}	6740~η_{100}	19
Pt_{SA}-NiO/Ni	26	86	27	20600~η_{100}	5.71~η_{50}	20
Pt_1/N-C	46	210	36.8	N/A	1.89~η_{50}	9
Pt_{SA}-$Co(OH)_2$	29	105	35	1600~η_{50}	N/A	21
IrCo@N-C	45	N/A	80	N/A	N/A	10
PtNi-O/C	40	105	79	7230~η_{70}	N/A	22
$Pt_{3.6}$Ni-S NWs	38	80~η_{80}	115	4370~η_{70}	N/A	23
$PtSe_2$/Pt	42	160~η_{60}	53	N/A	N/A	24
Rh@Pt_{2L}	5	63	30.5	32790~η_{100}	9.00~η_{100}	25
Ru@Ni-MOF	22	105	40	400~η_{100}	1.47~η_{100}	14
Pt/NiO@Ni/NF	34	110	39	532~η_{50}	2.01~η_{100}	26
**PtC**	**29**	**130**	**92**	**486~η_{50}**	**0.49~η_{50}**	**This**
**NGA-COF@Pt**	**17**	**52**	**24**	**5502~η_{50}**	**5.56~η_{50}**	**work**

Supplementary References

1 Zhang, J. Q. *et al.* Single platinum atoms immobilized on an MXene as an efficient catalyst for the hydrogen evolution reaction. *Nat. Catal.* **1**, 985-992 (2018).

2 Chen, H. *et al.* Promoting Subordinate, Efficient Ruthenium Sites with Interstitial Silicon for Pt-Like Electrocatalytic Activity. *Angew. Chem. Int. Ed.* **58**, 11409-11413 (2019).

3 Zhang, H. B. *et al.* Dynamic traction of lattice-confined platinum atoms into mesoporous carbon matrix for hydrogen evolution reaction. *Sci. Adv.* **4**, eaao6657 (2018).

4 Cherevko, S. *et al.* Dissolution of Noble Metals during Oxygen Evolution in Acidic Media. *Chemcatchem* **6**, 2219-2223 (2014).

5 Yang, Y. *et al.* Non-precious alloy encapsulated in nitrogen-doped graphene layers derived from MOFs as an active and durable hydrogen evolution reaction catalyst. *Energy Environ. Sci.* **8**, 3563-3571 (2015).

6 Zhuang, L. Z. *et al.* Sulfur-Modified Oxygen Vacancies in Iron-Cobalt Oxide Nanosheets: Enabling Extremely High Activity of the Oxygen Evolution Reaction to Achieve the Industrial Water Splitting Benchmark. *Angew. Chem. Int. Ed.* **59**, 14664-14670 (2020).

7 Yang, S. Z. *et al.* Covalent Organic Frameworks with Irreversible Linkages via Reductive Cyclization of Imines. *J. Am. Chem. Soc.* **144**, 9827-9835 (2022).

8 Ji, S. F. *et al.* Atomically Dispersed Ruthenium Species Inside Metal-Organic Frameworks: Combining the High Activity of Atomic Sites and the Molecular Sieving Effect of MOFs. *Angew. Chem. Int. Ed.* **58**, 4271-4275 (2019).

9 Fang, S. *et al.* Uncovering near-free platinum single-atom dynamics during electrochemical hydrogen evolution reaction. *Nat. Commun.* **11**, 1029 (2020).

10 Jiang, P. *et al.* Tuning the Activity of Carbon for Electrocatalytic Hydrogen Evolution via an Iridium-Cobalt Alloy Core Encapsulated in Nitrogen-Doped Carbon Cages. *Adv. Mater.* **30**, 1705324 (2018).

11 Liu, D. B. *et al.* Atomically dispersed platinum supported on curved carbon supports for efficient electrocatalytic hydrogen evolution. *Nat. Energy* **4**, 512-518 (2019).

12 Cheng, X. *et al.* Charge redistribution within platinum-nitrogen coordination structure to boost hydrogen evolution. *Nano Energy* **73**, 104739 (2020).

13 Wu, D. S. *et al.* Noble-Metal High-Entropy-Alloy Nanoparticles: Atomic-Level Insight into the Electronic Structure. *J. Am. Chem. Soc.* **144**, 3365-3369 (2022).

14 Deng, L. *et al.* Electronic Modulation Caused by Interfacial Ni‐O‐M (M=Ru, Ir, Pd) Bonding for Accelerating Hydrogen Evolution Kinetics. *Angew. Chem. Int. Ed.* **60**, 22276-22282 (2021).

15 Liu, Y. *et al.* Ru Modulation Effects in the Synthesis of Unique Rod-like Ni@Ni2P–Ru Heterostructures and Their Remarkable Electrocatalytic Hydrogen Evolution Performance. *J. Am. Chem. Soc.* **140**, 2731-2734 (2018).

16 Park, J. *et al.* Investigation of the Support Effect in Atomically Dispersed Pt on WO3−x for Utilization of Pt in the Hydrogen Evolution Reaction. *Angew. Chem. Int. Ed.* **58**, 16038-16042 (2019).

17 Peng, Y. *et al.* Hydrogen evolution reaction catalyzed by ruthenium ion-complexed graphitic carbon nitride nanosheets. *J. Mater. Chem. A* **5**, 18261-18269 (2017).

18 Ye, S. *et al.* Highly stable single Pt atomic sites anchored on aniline-stacked graphene for hydrogen evolution reaction. *Energy Environ. Sci.* **12**, 1000-1007, (2019).

19 Yang, Q. *et al.* Single Carbon Vacancy Traps Atomic Platinum for Hydrogen Evolution Catalysis. *Journal of the American Chemical Society* **144**, 2171-2178, (2022).

20 Zhou, K. L. *et al.* Platinum single-atom catalyst coupled with transition metal/metal oxide heterostructure for accelerating alkaline hydrogen evolution reaction. *Nat. Commun.* **12**, 3783 (2021).

21 Zhou, K. L. *et al.* Seamlessly conductive Co(OH)(2)tailored atomically dispersed Pt electrocatalyst with a hierarchical nanostructure for an efficient hydrogen evolution reaction. *Energy Environ. Sci.* **13**, 3082-3092 (2020).

22 Zhao, Z. P. *et al.* Surface-Engineered PtNi-O Nanostructure with Record-High Performance for Electrocatalytic Hydrogen Evolution Reaction. *J. Am. Chem. Soc.* **140**, 9046-9050 (2018).

23 Liu, Z. J. *et al.* Aqueous Synthesis of Ultrathin Platinum/Non-Noble Metal Alloy Nanowires for Enhanced Hydrogen Evolution Activity. *Angew. Chem. Int. Ed.* **57**, 11678-11682 (2018).

24 Wang, Z. *et al.* PtSe2/Pt Heterointerface with Reduced Coordination for Boosted Hydrogen Evolution Reaction. *Angew. Chem. Int. Ed.* **60**, 23388-23393 (2021).

25 Guo, Y. *et al.* Pt Atomic Layers Boosted Hydrogen Evolution Reaction in Nonacidic Media. *Adv. Energy Mater.* **12**, 2201548 (2022).

26 Chen, Z. J. *et al.* Highly Dispersed Platinum on Honeycomb-like NiO@Ni Film as a Synergistic Electrocatalyst for the Hydrogen Evolution Reaction. *ACS Catal.* **8**, 8866-8872 (2018).
