# Supplementary material for: Single-atom platinum with asymmetric coordination environment on fully conjugated covalent organic framework for efficient electrocatalysis
Source: Nat Commun. 2024 Mar 22;15:2556. doi: 10.1038/s41467-024-46872-x (PMC10960042; doi:10.1038/s41467-024-46872-x)
Supplement: Supplementary file 3 — Description of Additional Supplementary Files [file 41467_2024_46872_MOESM3_ESM.pdf]

Description of Additional Supplementary Files

File Name: Supplementary Data 1

Description: Atomic coordinates of the optimized computational structures models used for theoretical calculation in this paper.
